# Therapy of clinical stage IIA and IIB seminoma: a systematic review

**DOI:** 10.1007/s00345-021-03873-5

**Published:** 2021-11-15

**Authors:** Julia Heinzelbecker, Stefanie Schmidt, Julia Lackner, Jonas Busch, Carsten Bokemeyer, Johannes Classen, Annette Dieing, Oliver Hakenberg, Susanne Krege, Alexandros Papachristofilou, David Pfister, Christian Ruf, Hans Schmelz, Heinz Schmidberger, Rainer Souchon, Christian Winter, Friedemann Zengerling, Sabine Kliesch, Peter Albers, Christoph Oing

**Affiliations:** 1 Department of Urology and Paediatric Urology, Saarland University Medical Centre and Saarland University Faculty of Medicine, Homburg, Saar Germany; 2 UroEvidence@Deutsche Gesellschaft Für Urologie, Berlin, Germany; 3 Department of Urology, Vivantes Clinics am Urban, Berlin, Germany; 4 II. Medical Clinic and Polyclinic, University Hospital Hamburg-Eppendorf, Hamburg, Germany; 5 Department of Radiotherapy, Radiological Oncology and Palliative Medicine, St. Vincentius-Kliniken, Karlsruhe, Germany; 6 Clinic for Internal Medicine-Hematology and Oncology, Vivantes Clinics Am Urban, Berlin, Germany; 7 Urological Clinic and Polyclinic, University Hospital Rostock, Rostock, Germany; 8 KEM, Protestant Hospital Essen-Mitte, Clinic for Urology, Pediatric Urology and Urological Oncology, Essen, Germany; 9 Clinic of Radiotherapy and Radiation Oncology, University Hospital Basel, Basel, Switzerland; 10 Department of Urology, University Hospital Cologne, Cologne, Germany; 11 Department of Urology, Bundeswehrkrankenhaus (German Federal Armed Forces Hospital), Ulm, Germany; 12 Department of Urology, Bundeswehrkrankenhaus (German Federal Armed Forces Hospital), Koblenz, Germany; 13 Clinic and Polyclinic for Radiooncology and Radiotherapy, University Hospital Mainz, Mainz, Germany; 14 Department for Radiooncology, University Hospital Tübingen, Tübingen, Germany; 15 Urologie Neandertal (Regional Joint Practice), Erkrath, Germany; 16 Department of Urology, University Hospital Ulm, Ulm, Germany; 17 Centre of Reproductive Medicine and Andrology, Department of Clinical and Surgical Andrology, University Hospital Münster, Münster, Germany; 18 Department of Urology, University Hospital Düsseldorf, Düsseldorf, Germany; 19 Mildred Scheel Cancer Career Centre HaTriCs4, University Cancer Centre Hamburg, University Medical Centre Hamburg-Eppendorf, Hamburg, Germany

**Keywords:** Testicular cancer, Seminoma, CS IIA/B, Systematic review, Treatment, Toxicity

## Abstract

**Purpose:**

The optimal treatment for clinical stage (CS) IIA/IIB seminomas is still controversial. We evaluated current treatment options.

**Methods:**

A systematic review was performed. Only randomized clinical trials and comparative studies published from January 2010 until February 2021 were included. Search items included: seminoma, CS IIA, CS IIB and therapy. Outcome parameters were relapse rate (RR), relapse-free (RFS), overall and cancer-specific survival (OS, CSS). Additionally, acute and long-term side effects including secondary malignancies (SMs) were analyzed.

**Results:**

Seven comparative studies (one prospective and six retrospective) were identified with a total of 5049 patients (CS IIA: 2840, CS IIB: 2209). The applied treatment modalities were radiotherapy (RT) (*n *= 3049; CS IIA: 1888, CSIIB: 1006, unknown: 155) and chemotherapy (CT) or no RT (*n *= 2000; CS IIA: 797, CS IIB: 1074, unknown: 129). In CS IIA, RRs ranged from 0% to 4.8% for RT and 0% for CT. Concerning CS IIB RRs of 9.5%–21.1% for RT and of 0%–14.2% for CT have been reported. 5-year OS ranged from 90 to 100%. Only two studies reported on treatment-related toxicities.

**Conclusions:**

RT and CT are the most commonly applied treatments in CS IIA/B seminoma. In CS IIA seminomas, RRs after RT and CT are similar. However, in CS IIB, CT seems to be more effective. Survival rates of CS IIA/B seminomas are excellent. Consequently, long-term toxicities and SMs are important survivorship issues. Alternative treatment approaches, e.g., retroperitoneal lymph node dissection (RPLND) or dose-reduced sequential CT/RT are currently under prospective investigation.

**Supplementary Information:**

The online version contains supplementary material available at 10.1007/s00345-021-03873-5.

## Introduction

Testicular cancer (TC) is the most common solid malignancy in young men [1, 2]. Accounting for only 7% of all TC patients, clinical stage (CS) IIA/B seminoma, defined as disease spread to the retroperitoneal lymph nodes of up to 2 cm (CS IIA) or of more than 2 cm to up to 5 cm (CS IIB) in maximum diameter, is a rare disease [3, 4]. Established national and international TC guidelines recommend radiotherapy (RT) or chemotherapy (CT) for the treatment of CS IIA/B seminoma patients [5, 6]. However, high-level evidence to define the optimal treatment remains elusive [5, 6]. As survival rates of CS IIA/B patients are excellent and approach 100%, treatment-related acute and long-term toxicities are of considerable interest for shared treatment decision-making [7].

Here, we summarize the available evidence regarding the different treatment modalities of CS IIA/B seminoma including associated acute and long-term toxicities.

## Methods

This work is based on a systematic literature search that was conducted for the development of the first German clinical practice guideline [4, 5, 8].

We performed a systematic literature review in accordance with the preferred reporting items for systematic reviews and meta-analyses (PRISMA) guidelines [9]. The search was conducted in Medline (via Ovid) and the Cochrane Central Register of Controlled Trials (search period January 2010–February 2021). The detailed methods, including the search strategy, can be found in Suppl. 1.

Randomized controlled trials and comparative studies were considered if they included patients with IIA or IIB seminoma, who received additional treatment after surgical treatment of the primary tumor. For studies to be included, outcome data had to be displayed separately for these patient groups. Non-seminomatous disease as well as patients at other disease stages were excluded. Considered treatments were radiotherapy (RT), chemotherapy (CT), retroperitoneal lymph node dissection (RPLND) or combinations of these treatments. Our endpoints of interest were relapse rate (RR), overall (OS), cancer-specific survival (CSS), as well as adverse events [acute and late toxicities including treatment-related secondary malignancies (SM)].

## Results

**Fig. 1:**
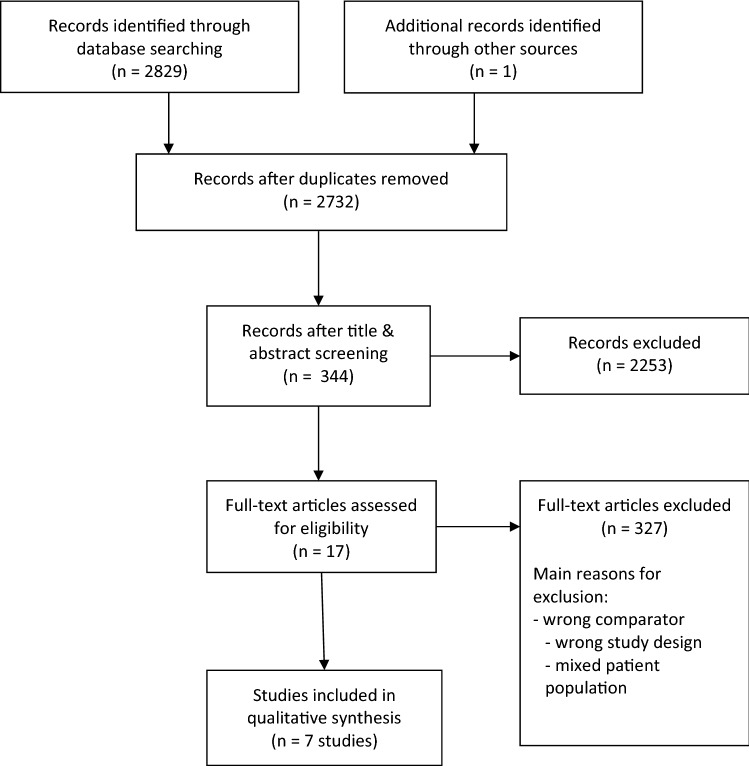
Flow chart of the study inclusion process for the systematic review according to PRISMA [9]

2830 records were identified through database searching or other sources. Seven comparative studies met our inclusion criteria. These were published between 2011 and 2017 [10–16] (see Table 1). There were one prospective and six retrospective trials including a total of 5049 assessable CS IIA/B patients (CS IIA: 2840; CS IIB: 2209). 3049 patients received RT (CS IIA: 1888; CS IIB: 1006; unknown: 155) and 2000 patients received CT or “no RT” (CS IIA: 797; CS IIB: 1074; unknown: 129) (unfortunately “no RT” was not further defined in the respective study [13], see below). Figure 1 displays the study identification process.

**Table 1:** Overview of the comparative studies on CS IIA/B seminoma patients included in the systematic review (a) patient and treatment characteristics, outcome parameters for relapse/survival and quality parameters (b) toxicities

(a)															
Author	Year (study period)	Type of study	*n*, overall (CS IIA/B)		Treatment modality	RT characteristics		CT regimen [courses]	Median FU in years (range; IQR)	Outcome				Time of reference	LoE
			n, CS IIA	n, CS IIB		Field	Dose (Gy)			RR (%)	RFS (%)	OS (%)	CSS (%)		
Tandstad	2011 (2000–2006)	Prospective cohort study SWENOTECA Swedish, Norwegian	1384^b^ (102)		RT (29; IIA); CT (73; IIA: 6, IIB: 67)	Paraaortic + iliac	27	EP [4]/BEP in large tumours [NA]	^b^5.2 (0.1–10.2) RT (IIA): 5.7 (2.4–9.5); CT (IIA): 5.2 (3.4–7.8); (IIB): 5.5 (2.1–9.3)	RT: 10.3 (IIA); CT: 0 (IIA; IIB) *p* = 0.01	IIA: RT: 88.7; CT: 100 IIB: RT: n.a; CT: 100	IIA, IIB: RT, CT: 100	IIA, IIB: RT, CT: 100	5	2b
			35	67											
Kollmannsberger	2011 (1999–2008)	Retrospective cohort study British Columbia Cancer Agency and Oregon Testis Cancer Program Canada, US	649^b^ (43)		RT (19); CT (24)	Paraaortic + iliac	35	EP [4]/BEP (3)	3.8 (0.2–9.2)	NA	IIA-C: RT: 91.7; CT: 95.5^b^	IIA-C: RT: 92.3; CT: 90.7^b^	NA	5	2b
			10	33											
Domont^c^	2013 (1980–2001)	Retrospective cohort study Patients at Institut Gustave Roussy, Villejuif, France	67^b^ (36)		RT (33; IIA: 5, IIB: 28); CT (3; IIB)	Paraaortic + iliac; M/SC	36	EP [4]/HOP [NA]/PVB [NA]/VAB [NA]	^b^9.4 (1–21)	RT: 24.2; IIA: 0, IIB: 28.6; CT: 0; IIA: n.a., IIB: 0	NA	IIA-C: 97 IIA-C: OS after relapse: RT: 82; CT: 88 p = 0.83	NA	5	2b
			5	31											
Ahmed	2015 (1988–2003)	Retrospective cohort study US SEER database	241		RT (136); no RT (105)	NA	NA	NA	10 (NA)	NA	NA	IIA: RT: 96, 96, 96; no RT: 88, 77, 77 *p* = 0.008 IIB: RT: 98, 96, 88; no RT: 90, 86, 86 *p* = 0.03	IIA: RT: 97, 97, 97; no RT: 96, 92, 92 IIB: RT: 98, 98, 98; no RT: 98, 96, 96	5, 10, 15	4^a^
			145	96											
Glaser	2016 (1998–2012)	Retrospective cohort study US National Cancer Data Base	2,437^b^ (1772)		RT (1,192; IIA: 750, IIB: 442); CT (580; IIA: 210, IIB: 370)	NA	IIA: 30.9; IIB: 35.5	NA	^b^5.4 (2.8–8.8)	NA	NA	IIA: RT: 99; CT: 93 IIB: RT: 95.2; CT: 92.4 HR: IIA: 0.22; *p* = 0.005	NA	5	2b
			960	812											
Paly	2016 (1998–2012)	Retrospective cohort study US National Cancer Data Base	1,885		RT (1,160; IIA: 780, IIB: 380); CT (725; IIA: 300, IIB: 425)	NA	IIA: 25.5, MBD: 4.5; IIB: 25.5, MBD: 10	NA	4.2 (IQR: 5.6)	NA	NA	IIA: RT: 99.4; CT: 91.2, *p* < 0.01 IIB: RT: 96.1; CT: 92.8 HR: IIA: 13.3 p < 0.01	NA	5	4^a^
			1,080	805											
Patel^c^	2017 (1988–2013)	Retrospective cohort study US SEER database	16,463^b^ (970)		RT (480; IIA: 324, IIB: 156); no RT (490; IIA: 281, IIB: 209)	NA	NA	NA	^b^7.4 (IQR: 3.3–12.1)	NA	NA	IIA: 95.8, 94.1, 94.1 IIB: 95.9, 91.7, 87.2 HR: RT vs. no RT: IIA: OS: 0.34 (0.14–0.8), *p* = 0.014	IIA: 98.1, 98.1, 98.1 IIB: 98, 97.2, 95.9	5, 10, 15	4^a^
			605	365											

(b)				
Author	Side effects			Risk of SM
	Acute toxicity: n (%)	Late toxicity: *n* (%)	Secondary malignancy: n (%)	
Domont^c^	^b^RT: Grade 1/2:	^b^RT: 4 (11%):	^b^RT in field: 3 (8%):	NA
	Nausea: 34 (92%)	Ototoxicity: 2 (5%)	Colorectal cancer: 1 (3%)	
	Diarrhea: 19 (51%)	Neuropathy: 2 (5%)	Duodenal cancer: 1 (3%)	
	Grade 3/4:	Fertility Disorders: NA	Medullary thyroid cancer: 1 (3%)	
	Nausea: 3 (8%)	Pulmonary fibrosis: NA	CT: 2 (7%):	
	CT: Grade 3/4:	Nephrotoxicity: 0 (0%)	Colorectal cancer: 1 (3%)	
	Neutropenia: 7 (22%)	CT: 8 (27%):	Esophageal cancer: 1 (3%)	
	Febrile neutropenia: 5 (15%)	Ototoxicity: 2 (7%)		
		Neuropathy: 1 (3%)		
		Fertility disorders: 2 (7%)		
		Pulmonary fibrosis: 1 (3%)		
		Grade 2 nephrotoxicity: 2 (7%)		
Patel^c^	NA	NA	^b^RT/no RT: overall nontesticular: 82 (0.9%)/30 (0.4%)	Relative risk of SM ^b^overall: 1.84 (1.6–2.1), *p* < 0.01; IIA: REF; IIB: 1.46 (CI 0.84–2.54), *p* = 0.175
			Oral/pharynx: 0 (0%)/1 (0.01%)	
			Gastrointestinal: 21 (0.2%)/10 (0.1%) Respiratory: 25 (0.3%)/8 (0.1%)	
			Urinary: 7 (0.1%)/0 (0%)	
			Gastrointestinal: 21 (0.2%)/10 (0.1%) Respiratory: 25 (0.3%)/8 (0.1%)	
			Lymphoma/leukemia: 15 (0.2%)/8 (0.1%)	
			Soft tissue/heart: 4 (0.04%)/2 (0.03%)	
			Skin: 4 (0.04%)/0 (0%)	
			Eye/orbit: 1 (0.01%)/0 (0%)	
			Nervous system: 2 (0.02%)/1 (0.01%)	
			Endocrine: 1 (0.01%)/0 (0%)	
			Myeloma: 1 (0.01%)/0	
			Miscellaneous: 1 (0.01%)/0 (0%)	

*BEP* bleomycin/etoposide/cisplatin, *CI* confidence interval, *CS* clinical stage, *CSS* cancer-specific survival, *CT* chemotherapy, *EP* etoposide/cisplatin, *FU* follow-up, *Gy* Gray, *HOP* ifosfamide, vincristine, cisplatin, *HR* hazard ratio, *IQR* interquartile range, *LoE* level of evidence, *M* mediastinal, *MDB* median boost dose, *n* number of patients, *NA* not announced, *n.a.* not applicable, *OS* overall survival, *PVB* cisplatin, vinblastine, bleomycin, *REF* reference group, *RFS* relapse-free survival, *RoB* risk of bias, *RR* relapse rate, *RT* radiotherapy, *SC* supraclavicular, *SEER* Surveillance, Epidemiology, and End Results Program, *SM* secondary malignancy, *SWENOTECA* Swedish and Norwegian Testicular Cancer Group, *US* United States, *VAB* vinblastine, cyclophosphamide, dactinomycin, bleomycin

^a^Level of evidence was downgraded due to study limitations from the risk of bias assessment

^b^Other stages as CS IIA/B are included

^c^Studies that also reported toxicities

Only three studies reported on the specific RT template (see Table 1a) [10–12]. The applied total cumulative radiation dose was reported in five studies ranging from 25.5 to 36 Gy [10–12, 14, 15]. Concerning CT, only three studies reported on CT regimens with EP (etoposide/cisplatin), EP or BEP (bleomycin/etoposide/cisplatin) and EP, HOP (ifosfamide, vincristine, cisplatin), PVB (cisplatin, vinblastine, bleomycin) or VAB (vinblastine, cyclophosphamide, dactinomycin, bleomycin) being used [10–12]. Of note, two studies used “no RT” as comparator to patients receiving RT, however did not further define “no RT” [13, 16]. Across studies, the median follow-up ranged between 3.8 and 10 years. Four studies were classified as level of evidence (LoE) 2b, three were downgraded to LoE 4, mainly because of missing information on outcome definition, assessment and statistical variance and no control for possible confounders. Details of the risk of bias assessment are given in Suppl. 2.

### Outcome for relapse and survival

In terms of outcomes, only two studies assessed RRs (see Table 1a) [10, 12]. One did not include CS IIA patients for CT, the other did not include CS IIB patients for RT. The overall RRs for RT were 10.2% and 24.2%. For CS IIA patients, a RR following RT of 0%–10.3% and for CS IIB patients of 28.6% was reported. For CT, both studies showed an overall RR of 0%. Tandstad et al. showed a statistically significant difference in the RRs of RT and CT (10.3% vs. 0%, *p* = 0.01) [10].

In terms of RFS, only two studies reported data (see Table 1a) [10, 11]. Kollmannsberger et al. studied CS II including CS IIC patients, with a 5-year RFS of 91.7% for RT and 95.5% for CT [11]. Tandstad et al. showed a 5-year RFS for CS IIA patients of 88.7% for RT and of 100% for CT (CS IIA/B) [10].

All studies reported on OS and three studies additionally on CSS (see Table 1a). Domont et al. showed for CS IIA/B patients a 5-year OS of 82% following RT versus 88% following CT [12]. Kollmannsberger et al. described a 5-year OS rate of 92.3% following RT and 90.7% following CT [11]. Patel et al. reported 5–15-year OS rates for CS IIA patients of 95.8%–94.1% and for CS IIB patients of 95.9%–87.2% [16]. The other four studies described treatment specific OS data for CS IIA and CS IIB patients [10, 13–15]. In CS IIA patients, the 5-year OS rates following RT were 96%–100% and for CT or “no RT” 88%–100%. Ahmed et al. and Paly et al. reported a significantly reduced OS after CT or “no RT” compared to RT in CS IIA seminoma patients (*p* = 0.008, *p* < 0.01) [13, 15]. In CS IIB patients, 5-year OS rates following RT were 95.2%–100% and following CT or “no RT” 90%–100%. Ahmed et al. reported reduced OS rates for CS IIB seminoma patients for “no RT” compared to RT-treated patients (*p* = 0.03) [13].

Regarding 5-year CSS, Tandstad et al. reported a 5-year CSS of 100% for CS IIA and CS IIB patients treated with RT as CT, respectively [10]. Patel et al. reported a 5–15-year CSS for CS IIA patients of 98.1%, respectively, and for CS IIB patients of 98%–95.9% [16]. Ahmed et al. reported a 5-year CSS of CS IIA patients of 97% after RT versus 96% for “no RT” and for CS IIB patients of 98% versus 98%, respectively [13].

Additionally, Glaser et al., Paly et al. and Patel et al. reported significantly reduced hazards ratios (HR) for death for RT in CS IIA patients, with HRs of 0.22–0.34 (*p* = 0.005, *p* = 0.014) with regards to RT [14, 16] and a significantly increased HR for death of 13.3 (*p* < 0.01) with regards to CT [15] (see Table 1a).

### Outcome for acute, late toxicities and secondary malignancies

Only two studies assessed toxicities (see Table 1b) [12, 16]. As reported by Domont et al., 92% of the RT patients experienced grade 1/2 nausea and 51% grade 1/2 diarrhea [12]. Grade 3/4 nausea occurred in 8% of RT patients. Grade 3/4 neutropenia occurred in 22% of CT patients and febrile neutropenia in 15%. Late toxicities were reported in 11% of RT patients and in 27% of CT patients with neuropathy, fertility disorders, pulmonary fibrosis and nephrotoxicity described for CT patients. In terms of SM, 8% of RT and 7% of CT patients developed a SM, which was colorectal, duodenal and medullary thyroid cancer in the RT and colorectal and oesophageal cancer in the CT groups.

Only Patel et al. reported on SMs, comparing patients who received RT to those who did not receive RT (median follow-up: 8.3 years) [16]. For the overall population (CS I–CS IIC), they reported a higher rate of SMs in patients who received RT (0.9% vs. 0.4%) with a significantly higher relative risk (RlR) of SMs in the RT group (RlR: 1.84). When adjusting for stage and age this result remained statistically significant for CS IA patients, only (see Table 1b).

## Discussion

RT and CT represent the most frequently applied and guideline-endorsed treatment options for CS IIA/B seminoma achieving excellent long-term outcomes [6, 8].

Concerning efficacy, in CS IIA RRs of 0–4.8% following RT at 30 Gy and of 0% following CT have been reported. For CS IIB patients, RRs after RT ranged from 9.5% to 21.1% at a 36 Gy cumulative dose and from 0 to 14.2% after CT. Thus, RT and CT seem equally effective in CS IIA, whereas CT tends to be more effective in CS IIB patients. Long-term outcomes are excellent with 5-year OS rates for CS IIA/B seminoma patients ranging from 90 to 100%, irrespective of the applied treatment. Nevertheless, survival data of CS IIA patients warrant attention as few studies, however with considerable risk of bias, found a reduced OS when CT was applied. The reasons for this so far remain unknown.

### RRs and RFS

Of the studies included for this systematic review, only the prospective study of Tandstad et al. included both treatments, RT and CT for CS IIA patients [10]. They reported a significantly higher RR following RT (11.3% vs. 0% following CT), but the applied cumulative radiation dose was only 27 Gy. Today, a radiation dose of 30 Gy is the guideline-endorsed standard dose for CS IIA patients based on several single-arm prospective trials reporting RRs of 0–4.8% [17–19].

Concerning CS IIB patients, only Domont et al. compared RT and CT outcomes [12]. The RR following RT was 28.6% versus 0% in the CT cohort, but only three patients of the study received CT. The applied cumulative radiation dose was 36 Gy in line with the nowadays recommended standard dose for CS IIB patients [17–19]. Reported RRs of single-arm prospective trials assessing RT efficacy both lower (9.5–11.1%), and higher RRs of up to 21.1% with a radiation dose of 36 Gy [17, 18, 20] and thus, the true RR following RT remains elusive but seems unanimously higher than following CT based on prospective study data.

Various other studies not included in this systematic review also addressed the treatment of CS IIA/B seminomas with different CT regimens and various cumulative RT doses [17–41] (see Suppl. 3). Today’s guideline-endorsed CT standard is three cycles of BEP [6, 8]. However, the CT regimens of the studies included in the systematic review were highly heterogeneous and do not represent the current standard of care. There exist several prospective single-arm trials evaluating different CT regimens [31, 34, 35]. Arranz Arija et al. evaluated up to four courses of EP in CS IIA/B seminoma patients and found a 3-year RFS of 91% [31]. Garcia-del-Muro et al. evaluated four courses of EP or three courses of BEP with RRs of 0% for CS IIA and 11.1% for CS IIB patients and a 5-year progression-free survival (PFS) of 100% and 87%, respectively [35]. Consequently, four cycles of EP may be considered in patients where Bleomycin has to be omitted, which is also the established approach for advanced metastatic germ cell tumors with a favorable IGCCCG risk profile [6, 8]. The optimal number of cycles of BEP remains controversial since retrospective analyses of either four or two cycles of BEP also reported a 0% RR [37, 38]. Three to four cycles of Carboplatin AUC7, is not equally effective compared to cisplatin-based combination CT with higher RRs reported in a prospective study by Krege et al. [34].

Taken together, RT and CT appear to be equally effective in terms of RRs in CS IIA. However, CT seems to have lower RRs in CS IIB patients than RT. This may be due to the given heterogeneity of CS IIB comprising small lesions with a diameter of just 2 cm to bulky nodal metastases with a diameter of up to 5 cm.

### OS and CSS

All studies included in this systematic review assessed OS as an endpoint and it is obvious that cure rates are unanimously high together with an extremely low rate of cancer-associated deaths [9–15]. Three studies reported on CSS [10, 13, 16]. However, only Tandstad et al. and Ahmed et al. discriminated for CS IIA or IIB and for RT or CT/”no RT”. 5-year CSS ranged from 97 to 100% in CS IIA patients treated with RT versus 96%–100% for CS IIA patients treated with CT/no RT [10, 13]. For CS IIB patients, CSS ranged from 98 to 100% for RT and from 98 to 100% for CT/no RT [10, 13]

Ahmed et al. found a statistically significant difference regarding OS for patients who did not receive RT [13]. Nevertheless, these results must be interpreted cautiously as there are certain limitations to the study, the most important being that “other approaches than RT” are not defined and that data on doses in the SEER database are missing (see Suppl. 2). The OS results of two other US National Cancer Database-based projects on CS IIA seminoma patients merit attention [14, 15]. Glaser et al. reported a significantly lower HR for death (HR: 0.22) for CS IIA patients treated with RT, with a 5-year OS of 99% (RT) compared to 93% (CT) [14]. This advantage even persisted on a propensity-adjusted multivariate analysis. However, neither CT regimens nor numbers of applied courses of CT were documented reflecting a substantial source of bias. In line with these results, Paly et al. reported a significantly higher HR of death (HR: 13.3) for CS IIB patients treated with CT, with a 5-year OS of 91.2% (CT) and 99% (RT) [15]. However, the study of Paly et al. also has major limitations due to the limited availability of only 80% of the data on radiation dose and lacking information about CT details. Nevertheless, both results are corroborated by a study of Patel et al. that assessed the SEER database and found a reduced HR for death (HR: 0.34) in CS IIA patients who received RT [16]. Again, also in this study, outcomes were not defined and there was lacking information on treatment details due to the limited data collection within SEER.

With regards to long-term survival, the risk of SM is of interest. Groot et al. evaluated cause-specific mortality among testicular cancer (TC) patients in a large multicentre cohort study with a 17.6-year median follow-up [42]. They found RT and CT to be associated with increased SM-related mortality. However, only the receipt of CT was additionally associated with increased standard mortality ratios (SMR) for ischemic heart disease (IHD) and respiratory disease. Though, when considering RT doses at > 26–32 Gy and > 32–36 Gy or platinum dosage < 400 mg/m^2^, the current standard doses applied in CS IIA/B seminoma patients, both RT and CT were associated with elevated SM mortality (HR: 1.98; 2.55), but CT was no longer associated with IHD or respiratory disease mortality [42].

### Alternative treatment approaches

There exist several studies on the combination of sequential CT and RT and on surgery by RPLND for the treatment of CS IIA/B seminoma [21–25, 36, 41]. However, none of them met the prespecified inclusion criteria of our performed systematic review. Patterson et al. evaluated the value of adding Carboplatin to RT on the basis of the SEER database [22]. They reported a RR for CS IIA patients of 7% (CT + RT) versus 13% (RT) and for CS IIB patients of 5% (CT + RT) versus 26% (RT), respectively. 5-year OS rates for CS IIA patients were 91.7% (CT + RT) versus 95.3% (RT) and for CS IIB patients 100% (CT + RT) versus 93.9% (RT) [22]. Horwich et al. reported on a pilot study of 51 CS IIA/B patients who were treated with the combination of a single cycle of Carboplatin CT followed by RT with reduced dose and extent of the radiation fields [21]. After a median follow-up of 4.6 years they reported no relapses. The prospective single-arm phase II SAKK 01/10 trial evaluated 3-year PFS in CS IIA/B seminoma patients treated with one cycle of Carboplatin AUC7 followed by reduced-field involved-node RT with 30 Gy in CS IIA and 36 Gy in CS IIB patients [43–45]. After a median follow-up of 4.5 years, 3-year PFS was 93.7% (CI 85.5–98.5%) in CS IIA and 95.2% (CI 85.2–96.4%) in CS IIB patients, respectively. There were no cancer-specific deaths [45]. Additionally, the single-arm phase II SAKK 01/18 (NCT03937843) trial evaluates 3-year PFS in seminoma patients who receive one cycle of Carboplatin AUC7 (CS IIA) or EP (CS IIB) followed by reduced-field involved-node RT with 24 Gy (CS IIA) or 30 Gy (CS IIB).

Surgical approaches by RPLND for CS IIA/B seminomas have also been reported on. Warszawski et al. reported a RR for CS IIA patients of 0% (RPLND) versus 10% (RT) and for CS IIB patients of 67% (RPLND) versus 20% (RT) [25]. Subsequently, several prospective trials assessed the efficacy of RPLND in CS IIA/B seminomas in an attempt to avoid CT- and RT-related, potentially life-threatening late toxicities [46, 47]. An interim analysis of the prospective phase II PRIMETEST trial, which included 22 CS IIA/B seminoma patients with a mean tumor size of 2.6 cm, displayed an overall 23% RR at a mean follow-up of 24 months. All patients remained relapse free after salvage treatment so far [41]. Preliminary results of the prospective phase II SEMS trial have also been published recently. Here, 55 CS IIA/B seminoma patients underwent RPLND. After a median follow-up of 24 months an overall RR of 18% was reported. The 2-year RFS rate was 85% and the 2-year OS rate 100% [48]. Both trials concluded that RPLND can be a therapeutic option as first-line treatment in early-stage metastatic seminoma. A caveat may be the so far limited follow-up period, as seminomas may relapse later than just two years from surgery.

### Toxicities

Only two studies of our systematic review report on treatment-associated toxicities and thus, evidence on treatment-related sequelae in CS IIA/B seminoma patients is scarce [12, 16]. Nausea and diarrhea are typical immediate side effects of RT [12, 17, 19], while nausea/vomiting, alopecia and transient bone marrow suppression are commonly related to platinum-based CT [31, 34, 35, 37] (see Suppl. 4). Treatment-related adverse events of RPLND comprise complications such as lymphocele, chylous ascites or ileus [23, 25, 48] (see Suppl. 4).

Concerning *long-term side effects* other than SMs, gastroesophageal reflux disease, sexual dysfunction, hypogonadism, diabetes and coronary artery disease have been described for RT [22, 33, 39, 49–51] and ototoxicity, neurotoxicity, nephrotoxicity and fertility disorders for CT [7, 12] (see Suppl. 4). A typical long-term side effect of RPLND is retrograde ejaculation; however, in modified template resections, as recommended for CSIIA/B seminoma patients, this risk can be minimized [48].

The most important, hence potentially life-threatening treatment-related long-term toxicities are *cardiovascular disease (CVD)* and *SMs* [7]. In terms of CT, an association with cardiovascular disease during the first year after CT has been described [51, 52]. Lauritsen et al. found that the risk of CVD thereafter decreased to normal levels. However, after ten years from diagnosis, there was an increasing risk for myocardial infarction and cardiovascular fatalities [51]. Nevertheless, it remains questionable if an association also exists for lower cumulative doses of CT as applied in CS IIA/B seminoma patients as Groot et al. found CT at dosages < 400 mg/m^2^ no longer to be associated with ischemic heart disease [42]. Additionally, Haugnes et al. found an increased odds for having intermediate/high risk cardiovascular morbidity or predicted mortality only in patients treated at platinum dosages > 850 mg/m^2^ [53].

SMs have been described as long-term adverse events for both CT and RT among TC patients [7]. Travis et al. report a cumulative risk of developing a secondary solid cancer for a 20-year-old seminoma patient of almost 50% by the age of 75 [54]. There is growing discussion on the amount of harm by the different therapies applied to TC patients accounting for solid SMs, whereas the risk of leukemia is typically ascribed to etoposide-based combination CT [7].

The impact of either CT or RT on SM risk remains controversial. Both studies of the systematic review reporting on toxicities, reported on SM. While Domont et al. found comparable amounts of SMs in in a very limited number of patients, Patel et al. reported a significantly higher risk of SMs following RT. However, after adjusting for stage, this remained significant for CS 1A seminoma patients only [12, 16]. Other reports on SM in CS IIA/B seminomas are mostly from single-arm RT studies reporting on in-field solid SMs mostly affecting the gastrointestinal (GI) and urinary tract organs (see Suppl. 4) [19, 22, 29, 33, 36, 38–40, 49]. Reports from CT or RPLND studies on the risk of SM specifically in CS IIA/B seminomas are scarce (see Suppl. 4) [22, 34, 36].

It, thus, turns out that both RT and CT are associated with increased standard incidence ratios (SIR) of various cancers, whereas there seems to be no risk of solid SMs after surgery alone, except for soft tissue sarcoma [55]. The receipt of CT was associated with an increased risk for solid SMs (HR: 2.4), colorectal (HR: 3.9) and non-colorectal GI SMs (HR: 5.0) [55]. However, at dosages of < 400 mg/m^2^ platinum, there was no statistically significant difference [55]. On the contrary, infradiaphragmatic RT at a cumulative dose of > 26–32 Gy and > 32–36 Gy was still associated with a higher solid SM risk (HR 2.4; 2.5), especially within the infradiaphragmatic regions (HR 3.4; 3.9) [55]. These results are in line with earlier reports of Travis et al. [54]. Among mainly RT-treated seminoma patients, increased SIR for SMs were restricted to infradiaphragmatic sites, with small intestine (SIR: 8.9), pancreatic (SIR: 4.4) and urinary bladder cancers (SIR: 3.4) [54].

Regarding SM mortality, Groot et al. found RT to be associated with a higher SM-related mortality especially due to colorectal, pancreatic and urologic malignancies, whereas CT was associated with a higher SM-related mortality from lung, colorectal, non-colorectal GI malignancies and leukemia [42]. Even at the currently applied doses in CS IIA/B seminomas, RT and CT both were associated with higher SM mortality [42].

There are several limitations to our systematic review. First, most of the studies were retrospective analyses, including data partly derived from the 1980s onwards. Second, level of evidence was mainly downgraded because missing information on outcome definition and assessment, missing information on statistical variance, and/or no control for possible confounders. Third, case numbers were mostly small and not solely including CS IIA/IIB patients. Fourth, treatment data, which are essential for the evaluation of treatment effects and harms, were rarely reported. As a result, essential treatment details were lacking, which did hamper data interpretation and therefore, we decided to not conduct a meta-analysis. Fifth, treatment regimens and diagnostic techniques have changed over time and do not reflect the various approaches assessed in the included studies anymore. This adds to the heterogeneity of identified data.

## Conclusion

Long-term outcomes of CSIIA/B seminoma patients are excellent and RT and CT represent equally effective treatment options especially for CS IIA patients, whereas in CS IIB seminomas, CT may be beneficial in terms of reduced RRs. Surgical and sequential approaches of CT and RT hold the promise to change the treatment landscape in the future. Shared decision-making with patients should be informed by the so far equal efficacy of RT and CT based on relatively low evidence. Particular attention should be paid on treatment-related side effects.

## Supplementary Information

Below is the link to the electronic supplementary material.

Supplementary file1 Suppl. 1: Detailed methods of the systematic review including the search strategy. (DOCX 21 KB)

Supplementary file2 Suppl. 2: Source of funding, conflict of interest and reasons for the classification of the risk of bias of the studies on CS IIA/B seminoma patients included in the systematic review. (DOCX 17 KB)

Supplementary file3 Suppl. 3: Patient, treatment characteristics and outcome parameters for relapse and survival of studies on CS IIA/B seminoma patients not included in the systematic review. (DOCX 45 KB)

Supplementary file4 Suppl. 4: Toxicities in CSIIA/B seminoma patients according to treatment. (DOCX 39 KB)
