# Supplementary material for: Therapy of clinical stage IIA and IIB seminoma: a systematic review
Source: World J Urol. 2021 Nov 15;40(12):2829–41. doi: 10.1007/s00345-021-03873-5 (PMC9712301; doi:10.1007/s00345-021-03873-5)
Supplement: Supplementary file 1 — Supplementary file1 Suppl. 1: Detailed methods of the systematic review including the search strategy. (DOCX 21 KB) [file 345_2021_3873_MOESM1_ESM.docx]

*Systematic literature search*

We performed a systematic literature review in the biomedical databases Medline (via Ovid) and the Cochrane Central Register of Controlled Trials (search period January 2010 to February 2021 by consensus of the authors as hardly no comparative studies have been published before 2010). Our search was limited to full text publications and to those written in English and German language. We considered randomized controlled trials and prospective and retrospective observational comparative studies. We also hand-searched the reference lists of included studies to determine additional, potentially relevant studies. Case reports, case series, editorials, comments and conference abstracts were excluded. An additional search for unpublished data and ongoing studies was conducted in the clinical trial registers Clinical Trials and International Clinical Trials Register Platform [1, 2].

Randomized controlled trials and comparative studies were considered if they included patients with proven IIA or IIB seminoma, who received adjuvant treatment after inguinal orchiectomy. For studies to be included, outcome data had to be displayed separately for these patient groups. Non-seminomatous disease as well as patients at other disease stages were excluded. Considered treatments were radiotherapy (RT), chemotherapy (CT), retroperitoneal lymph node dissection (RPLND) or combinations of these treatments. Our endpoints of interest were relapse rate (RR), overall (OS), cancer-specific survival (CSS), as well as adverse events (acute and late toxicities and treatment-induced secondary malignancies (SM)). However, missing reporting of certain outcome was not an exclusion criterion.

*Literature screening, data extraction and quality assessment*

One review author screened the titles and abstracts and afterwards the full texts of the retrieved references and determined the relevance for inclusion. For included studies, one author extracted relevant data in evidence tables. The risk of bias of included studies was appraised by one author using the SIGN checklist for cohort studies [3, 4]. The level of evidence was rated according to the Oxford criteria [5]. During all this process, another review author was involved in each of the above-mentioned steps and a consensus was reached by discussion in case of uncertainties.

1. Home - ClinicalTrials.gov. https://clinicaltrials.gov/. Accessed 9 Feb 2021

2. International Clinical Trials Registry Platform (ICTRP). https://www.who.int/clinical-trials-registry-platform. Accessed 9 Feb 2021

3. Scottish Intercollegiate Guidelines Network (SIGN). Checklists for cohort studies. In: SIGN. https://testing36.scot.nhs.uk. Accessed 14 May 2021

4. Shea BJ, Grimshaw JM, Wells GA, et al (2007) Development of AMSTAR: a measurement tool to assess the methodological quality of systematic reviews. BMC Med Res Methodol 7:10. https://doi.org/10.1186/1471-2288-7-10

5. Oxford Centre for Evidence-Based Medicine: Levels of Evidence (March 2009) — Centre for Evidence-Based Medicine (CEBM), University of Oxford. https://www.cebm.ox.ac.uk/resources/levels-of-evidence/oxford-centre-for-evidence-based-medicine-levels-of-evidence-march-2009. Accessed 11 Feb 2021

**Suppl. 1**: Detailed methods of the systematic review including the search strategy

Exemplarily, the search strategy for the Medline search is displayed:

Ovid MEDLINE(R) and Epub Ahead of Print, In-Process & Other Non-Indexed Citations

| 21 | limit 20 to yr="2010-current" |
| --- | --- |
| 20 | limit 19 to (english or german) |
| 19 | limit 18 to male |
| 18 | 16 not 17 |
| 17 | cancer-testis antigen*.tw. |
| 16 | 14 not 15 |
| 15 | exp animals/ not humans.sh. |
| 14 | 8 not 13 |
| 13 | 9 or 10 or 11 or 12 |
| 12 | note/ or editorial/ or letter/ or Comment/ or news/ or opinion/ |
| 11 | conference abstract.pt. or congresses as Topic/ |
| 10 | case reports/ or case reports.tw. |
| 9 | "review"/ |
| 8 | 1 or 2 or 3 or 4 or 5 or 6 or 7 |
| 7 | non*seminom*.tw. |
| 6 | (germinomatous or non*germinomatous).tw. |
| 5 | germ cell tumor.tw. |
| 4 | ((testicular or testis or testes) adj2 (tumor* or cancer* or carcinoma* or tumour* or neoplasm* or neoplasia)).tw. |
| 3 | *testicular neoplasms/ |
| 2 | seminom*.tw. |
| 1 | exp seminoma/ |
