# Supplementary material for: Therapy of clinical stage IIA and IIB seminoma: a systematic review
Source: World J Urol. 2021 Nov 15;40(12):2829–41. doi: 10.1007/s00345-021-03873-5 (PMC9712301; doi:10.1007/s00345-021-03873-5)
Supplement: Supplementary file 2 — Supplementary file2 Suppl. 2: Source of funding, conflict of interest and reasons for the classification of the risk of bias of the studies on CS IIA/B seminoma patients included in the systematic review. (DOCX 17 KB) [file 345_2021_3873_MOESM2_ESM.docx]

| **Author** | **Department** | **Source of funding, CoI** | **RoB** | **Reasons** |
| --- | --- | --- | --- | --- |
| Tandstad | Oncology | Swedish Cancer Society; Gunnar Nilsson Foundation for Cancer Research; Nordic Cancer Union  No CoI | Acceptable | - No mention of confounding |
| Kollmannsberger | Medical Oncology; Radiation Oncology; Urology | No CoI | Acceptable | - No indication if or how measures were managed, regarding outcome assessment - No mention of confounding - No confidence intervals provided |
| Domont | Medicine; Biostatistics; Radiotherapy | NA | Acceptable | - No indication if or how measures were managed, regarding outcome assessment - No mention of confounding - No confidence intervals provided - No separate analysis for tumour stages IIA, IIB and IIC |
| Ahmed | Radiation Oncology | NA | Not acceptable | - Outcomes were not a priori defined - No indication if or how measures were managed, regarding outcome assessment - No mention of confounding - No confidence intervals provided - Comparison of therapy is limited due to missing data of doses in the SEER database and “other approaches” is not defined |
| Glaser | Radiation Oncology; Epidemiology | NA | Acceptable | - lacking information about treatment details |
| Paly | Radiation Oncology | American Cancer Society intramural research funding  No CoI | Not acceptable | - limited availability of 80% of the data on radiation dose - lacking information about treatment details for chemotherapy |
| Patel | Urology | No CoI | Not acceptable | - Outcomes were not defined - lacking information about treatment details |

**Suppl. 2: Source of funding, conflict of interest and reasons for the classification of the risk of bias**

CoI= conflict of interest; NA= not announced; RoB= risk of bias; SEER= Surveillance, Epidemiology, and End Results Program
