# Supplementary material for: Therapy of clinical stage IIA and IIB seminoma: a systematic review
Source: World J Urol. 2021 Nov 15;40(12):2829–41. doi: 10.1007/s00345-021-03873-5 (PMC9712301; doi:10.1007/s00345-021-03873-5)
Supplement: Supplementary file 3 — Supplementary file3 Suppl. 3: Patient, treatment characteristics and outcome parameters for relapse and survival of studies on CS IIA/B seminoma patients not included in the systematic review. (DOCX 45 KB) [file 345_2021_3873_MOESM3_ESM.docx]

| **Author** | **Year (time frame)** | **Type of study** | **n, overall** | | | **Treatment modality** | **RT characteristics** | | **CT regimen [courses], (n)** | **RPLND mode** | **Median FU in years (range; IQR)** | **Outcome** | | | | **Time of reference** |
| --- | --- | --- | --- | --- | --- | --- | --- | --- | --- | --- | --- | --- | --- | --- | --- | --- |
|  |  |  | **n, CS IIA** | | **n, CS IIB** |  | **Field** | **Dose in Gy, (n)** |  |  |  | **RR (%)** | **RFS (%)** | **OS (%)** | **CSS (%)** |  |
| Schmidberger^c^ | 1997 (1991-1994) | Prospective | 58 | | | RT | Paraaortic + iliac | IIA: 30  IIB: 36 | n.a. | n.a. | 3.1 (NA) | IIA: 0, IIB: 10.5 | IIA: 100  IIB: 87.4 | IIA: 100  IIB: 100 | NA | 2 |
|  |  |  | 39 | | 19 |  |  |  |  |  |  |  |  |  |  |  |
| Warszawski | 1997  (1975-1991) | Retrospective | 161* (28) | | | RT (15; IIA: 10; IIB: 5); RPLND (13; IIA: 7; IIB: 6) | Paraaortic + iliac (10, IIA), Paraaortic + bilateral iliac (5, IIB) | IIA: 33 IIB: 37,7 | n.a. | bilateral, no routine pelvic ln | NA | RT: IIA: 10, IIB: 20;  RPLND: IIA: 0, IIB: 67 | NA | NA | NA | NA |
|  |  |  | 17 | | 11 |  |  |  |  |  |  |  |  |  |  |  |
| Warde | 1998 (1981-1993) | Retrospective | 99* (69) | | | RT (64; IIA: 40; IIB: 24); CT (5; IIA: 1; IIB: 4) | Paraaortic + iliac | IIA: 25  IIB: at least 30 | EP [3-6]/BEP/VAB** | n.a. | *6.7 (1-14) | IIA: 10, IIB: 12.5 | IIA/B: 89 | NA | NA | 5 |
|  |  |  | 41 | | 28 |  |  |  |  |  |  |  |  |  |  |  |
| Bauman | 1998 (1950-1995) | Retrospective | 212* (36) | | | RT | Paraaortic + iliac, Paraaortic + bilateral iliac, Scrotal Boost, Inguinal boost, M + SC | 30 | n.a. | n.a. | NA | RT: IIA: 8, IIB: 10 | NA | NA | NA | NA |
|  |  |  | 26 | | 10 |  |  |  |  |  |  |  |  |  |  |  |
| Weissbach | 1999 (1986-1991) | Prospective | 803* (82) | | | RT | Paraaortic + iliac | 36 | n.a. | n.a. | *3 (NA) | RT: IIA: 2.2, IIB: 21.1 | IIA: 94, IIB: 77 | IIA: 96, IIB: 83 | NA | 5, 10 |
|  |  |  | 44 | | 38 |  |  |  |  |  |  |  |  |  |  |  |
| Bamberg^c^ | 1999 (1991-1994) | Prospective | 756* (86) | | | RT | Paraaortic + iliac | IIA: 30  IIB: 36 | n.a. | n.a. | 4.6 (0.3-6.7) | RT: IIA: 4.8, IIB: 11.1 | IIA/B: 92.5 | NA | IIA/B: 100 | 4 |
|  |  |  | 65 | | 21 |  |  |  |  |  |  |  |  |  |  |  |
| Zagars | 2001 (1960-1999) | Retrospective | 73* (44) | | | RT | Paraaortic + iliac + M (16), Paraaortic + iliac (13), Paraaortic + iliac + SC (15) | IIA: <30 (2), ≥30 (4)  IIB: <30 (7), ≥30: (31) | n.a. | n.a. | *9.3 (1.2-27.4) | RT: IIA: 0, IIB: 13 | IIA: 100, IIB: 87 | *93 | *100 | 6 |
|  |  |  | 6 | | 38 |  |  |  |  |  |  |  |  |  |  |  |
| Patterson | 2001 (1970-1996) | Retrospective | 113 | | | RT (80; IIA: 46; IIB: 34); CT + RT (33; IIA: 14; IIB: 19) | Paraaortic + iliac, Supra- + infradiaphragmatic | 30-45 | CT+RT: Carbo AUC 7 [1], (33) ; Carbo AUC 7 [2], (2); Carbo/Etoposid [1], (1) | n.a. | RT: 11.2 (0.5-25.8);  CT+RT: 4 (0.1-7.8) | RT: IIA: 13, IIB: 26;  CT+RT: IIA: 7, IIB: 5 | RT: IIA: 86.6, IIB: 72.9; CT+RT: IIA: 92.3, IIB: 100 | RT: IIA: 95.3, IIB: 93.9;  CT+RT: IIA: 91.7, IIB: 100 | NA | 5 |
|  |  |  | 60 | | 53 |  |  |  |  |  |  |  |  |  |  |  |
| Arranz Arija | 2001 (1994-1999) | Prospective | 64* (34) | | | CT | n.a. | n.a. | E400P: 20mg/m² Cisplatin, 100mg/m² Etoposid; 3w [up to 4] | n.a. | *2.8 (NA) | NA | IIA/B: 91 | IIA/B: 95 | NA | 3 |
|  |  |  | NA | | NA |  |  |  |  |  |  |  |  |  |  |  |
| Classen^c^ | 2003 (1991-1994) | Prospective | 87 | | | RT | Paraaortic + iliac | IIA: 30  IIB: 36 | n.a. | n.a. | 5.8 (0.3- 9.3), IIA/B | IIA: 3; IIB: 9.5 | IIA: 95.3; IIB: 88.9 | NA | IIA: 100; IIB: 100 | 6 |
|  |  |  | 66 | | 21 |  |  |  |  |  |  |  |  |  |  |  |
| Chung | 2004 (1981-1999) | Retrospective | 126* (87) | | | RT (79; IIA: 49; IIB: 30); CT (8; IIA: 1; IIB: 7) | Paraaortic + iliac (+/- contralateral) | 25 (35); 35 (44) | EP [3-6], BEP [4], VAB [3]** | n.a. | *RT: 8.9; CT: 7.3 (0.6-20.7) | RT: IIA: 5.1, IIB: 10;  CT: IIA: 0, IIB: 14.2 | RT: IIA: 91.7, IIB: 89.7;  CT: IIA: 100, IIB: 83.3 | NA | IIA: 95.9; IIB: 100 | 5 (RFS); 5,10 (OS,CSS) |
|  |  |  | 50 | | 37 |  |  |  |  |  |  |  |  |  |  |  |
| Garcia-Serra | 2005 (1966-2000) | Retrospective | 73* (16) | | | RT | Paraaortic + iliac (+/- M, SC (25)) | median 25Gy (range 20-35) | n.a. | n.a. | *15 (NA) | *4.1 | NA | NA | *96 | 20 |
|  |  |  | 14 | | 2 |  |  |  |  |  |  |  |  |  |  |  |
| Krege | 2006 (1995-2001) | Prospective | 108 | | | CT | n.a. | n.a. | Carbo AUC 7: IIA: [3]; IIB: [4] | n.a. | 2.3 (0.1- 5.7) | 18; IIA: 16; IIB: 11 | NA | 99 | 100 | NA |
|  |  |  | 51 | | 57 |  |  |  |  |  |  |  |  |  |  |  |
| Mezvrishvili | 2006 (1997-2002) | Retrospective | 14* (4) | | | RPLND | n.a. | n.a. | n.a. | unilateral template, NS | *4.7 (2.4- 7.7) | *0 | NA | NA | NA | NA |
|  |  |  | 4 | | n.a. |  |  |  |  |  |  |  |  |  |  |  |
| Garcia del Muro | 2008 (1994-2003) | Prospective | 72 | | | CT | n.a. | n.a. | EP [4], BEP [3] | n.a. | 6 (NA) | IIA: 0; IIB: 11.1 | ^a^IIA: 100; IIB: 87 | 95 | NA | 5 |
|  |  |  | 18 | | 54 |  |  |  |  |  |  |  |  |  |  |  |
| Detti | 2009 (1965-2005) | Retrospective | 106* (102) | | | RT (89; IIA: 83; IIB: 6); CT+RT: (13; IIB) | Paraaortic + iliac: 37; Paraaortic + iliac + SC: 23; Paraaortic + iliac + M + SC: 42 | 30 (16); 30–36 (73); 40 (13) | BEP [3], (9), PVB [3], (4) | n.a. | *21 (1.2-41) | NA | IIA: 94; IIB: 72.5 | NA | IIA: 98.8; IIB: 79 | 5 (RFS); 5, 10 (CSS) |
|  |  |  | 83 | | 19 |  |  |  |  |  |  |  |  |  |  |  |
| Giannis | 2009 (1995-2007) | Retrospective | 52* (24) | | | CT | n.a. | n.a. | BEP [4] | n.a. | *5.8 (1.3-15.6) | *0 | NA | NA | NA | NA |
|  |  |  | 2 | | 22 |  |  |  |  |  |  |  |  |  |  |  |
| Pichler | 2012 (1996-2005) | Retrospective | 15 | | | CT | n.a. | n.a. | BEP [2] | n.a. | 5 (1.1- 15.4) | 0 | NA | NA | NA | NA |
|  |  |  | 5 | | 11 |  |  |  |  |  |  |  |  |  |  |  |
| Hallemeier | 2013 (1974-2007) | Retrospective | 52* (31) | | | RT | Paraaortic + iliac (M + SC until 1989) | 31 | n.a. | n.a. | *19 (0.4-37) | IIA: 12.5; IIB: 42.9 | IIA: 83; IIB: 54 | IIA: 96; IIB: 83 | IIA: 100; IIB: 83 | 10 |
|  |  |  | 24 | | 7 |  |  |  |  |  |  |  |  |  |  |  |
| Sridharan^b^ | 2013 (1995-2010) | Retrospective | 106 | | | RT (87; IIA: 58; IIB: 30); CT (18; IIB) | Paraaortic + iliac | 35 | EP [4], BEP [3] | n.a. | 6.1 (NA) | IIA: 8.6 (RT); IIB: 8.3 (RT/CT);  RT: 10 (IIA/B); CT: 5.6 (IIB) | 91 | 100 | NA | 5 |
|  |  |  | 58 | | 48 |  |  |  |  |  |  |  |  |  |  |  |
| Horwich | 2013 (1996-2011) | Retrospective | 51 | | | CT + RT | Paraaortic + iliac: 12; paraaortic: 38, paraaortic + ipsi: 1 | 30 (39); 35 (12) | Carbo AUC 7 [1] | n.a. | 4.6 (0.7-12.6) | 0 | 100 | NA | NA | 5 |
|  |  |  | 19 | | 32 |  |  |  |  |  |  |  |  |  |  |  |
| Stein | 2014 (1971-2010) | Retrospective | 24 | | | RT | Paraaortic + iliac | 22.5-25/5-10 | n.a. | n.a. | 7 (3.5-23.5) | NA | 88 | NA | NA | NA |
|  |  |  | 24 | | 0 |  |  |  |  |  |  |  |  |  |  |  |
| Hu | 2015 (2010-2014) | Retrospective | 4* (3) | | | RPLND | n.a. | n.a. | n.a. | Modified template, NS | *2.1 (NA) | *0 | NA | NA | NA | NA |
|  |  |  | 3 | | 0 |  |  |  |  |  |  |  |  |  |  |  |
| Albers^b^ | 2019 (2014-2018) | Prospective | 22 (17) | | | RPLND | n.a. | n.a. | n.a. | NA | *2 (0.1-4.3) | *23 | NA | NA | NA | NA |
|  |  |  | 4 | | 13 |  |  |  |  |  |  |  |  |  |  |  |
| Daneshmand^b^ | 2021 (NA) | Prospective | 55 | | | RPLND | n.a. | n.a. | n.a. | Open, modified template | NA | 18 | 84 | 100 | NA | 2 |
|  |  |  | NA | | NA |  |  |  |  |  |  |  |  |  |  |  |
| Papachristofilou^b^ | 2021 (2012-2018) | Prospective | 116 | | | CT + RT | Involved-node | IIA: 30  IIB: 36 | Carbo AUC 7 [1] | n.a. | 4.5 (0.8-8.1) | IIA: 2.2; IIB: 8.6 | ^a^93.7; IIA: 95.2, IIB: 92.6 | NA | NA | 3 |
|  |  |  | 46 | 70 | |  |  |  |  |  |  |  |  |  |  |  |

**Suppl. 3: Patient, treatment characteristics and outcome parameters for relapse and survival of studies on CS IIA/B seminoma patients not included in the systematic review.**

AUC= area under the curve; BEP= bleomycin/etoposid/cisplatin; Carbo= Carboplatin; CS= clinical stage; CSS= cancer specific survival; CT= chemotherapy; EP= etoposid/cisplatin; FU= follow-up; Gy= Gray; ipsi= ipsilateral; IQR= interquartile range; ln= lymph nodes; M= mediastinal; n= number of patients; NA= not announced; n.a.= not applicable; NS= nerve sparing; OS= overall survival; PVB= cisplatin, vinblastin, bleomycin; RFS= relapse free survival; RPLND= retroperitoneal lymph node dissection; RR= relapse rate; RT= radiotherapy; SC= supraclavicular; VAB= vinblastine, cyclophosphamide, dactinomycin, bleomycin; w= weekly; *other stages as IIA/B are included; **unclear what CT patients received which regimen and how many courses; ^a^progression free survival; ^b^only abstract available; ^c^results of a very similar patient collective at different follow-up endpoints
