# Supplementary material for: Therapy of clinical stage IIA and IIB seminoma: a systematic review
Source: World J Urol. 2021 Nov 15;40(12):2829–41. doi: 10.1007/s00345-021-03873-5 (PMC9712301; doi:10.1007/s00345-021-03873-5)
Supplement: Supplementary file 4 — Supplementary file4 Suppl. 4: Toxicities in CSIIA/B seminoma patients according to treatment. (DOCX 39 KB) [file 345_2021_3873_MOESM4_ESM.docx]

| **Author** | **Year**  **(time frame)** | **n, overall (CSIIA/B)** | **Treatment modality (n)** | **Treatment specification** | **Median FU in years (range; IQR)** | **Side-effects** | | |  |
| --- | --- | --- | --- | --- | --- | --- | --- | --- | --- |
|  |  |  |  |  |  | **Acute toxicity: n (%)** | **Late toxicity: n (%)** | **Secondary malignancy: n (%)** | **Risk of SM** |
| Schmidberger^a^ | 1997 (1991-1994) | 58 | RT | Paraaortic + iliac | 3.1 (NA) | Grade 1/2:   - Nausea: 42 (72%) - Diarrhoea: 18 (31%) - Cutaneous: 9 (16%)   Grade 3/4:   - Nausea: 5 (9%) - Diarrhoea: 3 (5%) | Hyperpigmentation of skin: 1 (2%) | NA | NA |
| Warszawski | 1997 (1975-1991) | 161* (28) | RT (*98); RPLND (*63) | RT: Paraaortic + iliac, Paraaortic + bilateral iliac  RPLND: bilateral | NA | *RT: G4:   - Renal insufficiency requiring dialysis: 3 (2%) - Enterocolitis requiring surgical intervention: 1 (1%)   RPLND: G4:   - Ileus: 1 (2%) - Perforation: 1 (2%)   both requiring surgical intervention | NA | NA | NA |
| Bauman | 1998 (1950-1995) | 212* (36) | RT | Paraaortic + iliac, Paraaortic + bilateral iliac, Scrotal Boost, Inguinal boost, M + SC | NA | *Nausea/vomiting: 128 (60%)  Diarrhoea: 100 (47%)  Peptic ulcer disease: 9 (4%) | *Bowel toxicity: 3 (1%) | *Nontesticular: 6 (3%):   - Cholangiocarcinoma - Rectal cancer - Prostate cancer - Bladder cancer - Glioblastoma   Contralateral testicular tumour: 3 (1%) | NA |
| Bamberg^a^ | 1999 (1991-1994) | 756* (86) | RT | Paraaortic + iliac | 4.6 (0.3-6.7), IIA/B | Grade 1/2 (IIA/B):   - Nausea: 47 (55%) - Vomiting: 11 (13%) - Diarrhoea: 17 (20%) - Cutaneous: 10 (12%)   Grade 3/4 (IIA/B):   - Nausea IIA/B: 9 (10%) | ^A^none | *Nontesticular: NA  Contralateral testicular tumour: 1 (0.1%)  Contralateral: GCNIS 1 (0.1%) | NA |
| Patterson | 2001 (1970-1996) | 113 | RT (*80); CT+RT (*33) | Paraaortic + iliac, Supra- + infradiaphragmatic | RT: 11.2 (0.5-25.8); CT+RT: 4 (0.1-7.8) | RT: 64 (80%): nausea and/or epigastric discomfort and/or loose stools during RT  CT+RT: 28 (85%): NA | RT:   - Peptic ulcer disease: 4 (5%) - Chronic dyspepsia: 1 (1%) - Diarrhoea: 1 (1%) - L`hermittes: 2 (3%) - Herpes zoster: 2 (3%) - Radiation skin reaction: NA - Non-fatal myocardial infarction: 2 (3%)   CT+RT:   - Peptic ulcer disease: 2 (6%) - Chronic dyspepsia 1 (3%) - Radiation skin reaction: 2 (6%) - Tinnitus: 1 (3%) | RT: Nontesticular in field: 5 (6%):   - Bladder cancer: 1 (1%) - Pancreas cancer: 1 (1%) - Lung cancer: 1 (1%) - Renal cancer; 1 (1%) - Parasternal BCCA: 1 (1%)   CT: 0 | NA |
| Arranz Arija | 2001 (1994-1999) | 64* (34) | CT | E400P | *2.8 (NA) | *Grade 1/2: NA  Grade 3/4:   - Leucopenia: 21 (33%) - Febrile neutropenia: 6 (9%) - Anemia: 2 (3%) - Thrombocytopenia: 2 (3%) - Emesis: 2 (3%) - Mucositis: 2 (3%) - Neurotoxicity: 1 (2%) | none | NA | NA |
| Classen^a^ | 2003 (1991-1994) | 87 | RT | Paraaortic + iliac | 5.8 (0.3- 9.3), IIA/B | Grade 1/2:   - Nausea (IIA/B): 43 (65%)/ 14 (66%) - Diarrhoea (IIA/B): 16 (24%)/ 7 (34%) - Skin (IIA/B): 6 (9%)/ 5 (24%)   Grade 3/4:   - Nausea (IIA/B): 5 (8%)/ 2 (10%) - Diarrhoea (IIA/B): 4 (6%)/ 2 (10%) | Hyperpigmentation of skin: 1 (0.01%) | none | NA |
| Garcia-Serra | 2005 (1966-2000) | 73* (16) | RT | Paraaortic + iliac (+/- M, SC) | *15 (NA) | NA | ^B^Gastroesophageal reflux disease: 11 (27%)  ^B^Peptic ulcer disease: 1 (0.02%)  ^B^Intestinal necrosis: 1 (0.02%)  ^B^Sexual function alteration: 12 (29%)  ^B^Infertility: 5 (12%)d  ^B^Hypogonadism: 12 (29%)  ^C^Coronary artery disease: 6 (24%) | *Nontesticular: 6 (6%):   - CLL: 1 (0.01%) - Solid tumours: 5 (7%): - Metastatic CUP - HNSCC - Melanoma - Chondrosarcoma - Prostate cancer - Thyroid cancer - BCCA   Contralateral testicular tumour: 1 (1%) | NA |
| Krege | 2006 (1995-2001) | 108 | CT | Carbo AUC 7 | 2.3 (0.1- 5.7) | Grade 1/2:   - Anemia: 60 (56%) - Leukocytopenia: 46 (43%) - Thrombocytopenia: 33 (31%) - Creatinine: 3 (3%) - Nausea: 69 (64%) - Vomiting: 33 (31%) - Stomatitis: 5 (5%) - Ototoxicity: 3 (3%) - Infection: 8 (7%) - Alopecia: 6 (6%)   Grade 3/4:   - Anemia: 1 (1%) - Leukocytopenia: 4 (4%) - Thrombocytopenia: 9 (8%) - Nausea: 5 (5%) - Vomiting: 4 (4%) - Haematuria: 1 (1%) | NA | Rectal cancer: 1 (1%) | NA |
| Mezvrishvili | 2006 (1997-2002) | 14* (4) | RPLND | unilateral template, NS | *4.7 (2.4-7.7) | *Postoperative wound infection: 1 (7%); antegrade ejaculation: 0 (0%) | NA | NA | NA |
| Garcia del Muro | 2008 (1994-2003) | 72 | CT | EP, PEB | 6 (NA) | Grade 1/2:   - Granulocytopenia: 18 (25%) - Thrombocytopenia: 7 (10%) - Anemia: 3 (4%) - Vomiting 27 (38%) - Mucositis: 5 (7%) - Diarrhoea: 1 (1%) - Peripheral neuropathy: 14 (19%) - Rash: 3 (4%) - Ototoxicity: 2 (3%) - Alopecia: 72 (100%)   Grade 3/4:   - Granulocytopenia: 9 (13%) - Thrombocytopenia: 2 (3%) - Anemia 4 (6%) - Febrile neutropenia: 8 (11%) - Vomiting: 6 (8%) - Mucositis: 1 (2%) - Diarrhoea: 1 (2%) - Pulmonary toxicity: 1 (2%) | none | NA | NA |
| Detti | 2009 (1965-2005) | 106* (102) | RT; CT+ RT | RT: Paraaortic + iliac; Paraaortic + iliac + SC; Paraaortic + iliac + M + SC  CT: PEB, PVB | *21 (1.2-41) | *RT/CT+RT:  Nausea and/or epigastric discomfort and/or loose stools: 36 (35%) | *RT/CT+RT:   - Cardiovascular disease: 9 (9%): - Acute myocardial infarction: 5 (5%) - Angina pectoris: 4 (4%) | Nontesticular: 2 (2%):   - Prostate cancer: 1 (1%) - Colorectal cancer: 1 (1%)   Contralateral testicular tumour: 2 (2%) | NA |
| Giannis | 2009 (1995-2007) | 52* (24) | CT | PEB | *5.8 (1.3-15.6) | *Grade 1/2: NA  Grade 3/4:   - Anemia: 3 (6%) - Neutropenia: 7 (13%) - Thrombocytopenia: 2 (4%) - Fatigue: 2 (4%) - Nausea/vomiting: 7 (13%) - Diarrhoea: 1 (2%) - Neurotoxicity: 2 (4%) - Ototoxicity: 1 (2%) | NA | NA | NA |
| Pichler | 2012 (1996-2005) | 15 | CT | PEB | 5 (1.1-15.4) | NA | NA | Melanoma: 1 (7%) | NA |
| Hallemeier | 2013 (1974-2007) | 52* (31) | RT | Paraaortic + iliac; M/SC | *19 (0.4-37) | NA | *Major cardiac events: 10 (19%):   - Myocardial infarction: 7 (13%) - Valve replacement: 2 (4%) - Coronary artery stent placement: 1 (2%) | *Nontesticular: 5 (10%):   - Oesophageal cancer: 2 (4%) - Periampullary cancer: 1 (2%) - Retroperitoneal undifferentiated neoplasm: 1 (2%) - Papillary thyroid cancer: 1 (2%) | NA |
| Horwich | 2013 (1996-2011) | 51 | CT + RT | CT: Carbo AUC 7  RT: Paraaortic + iliac; paraaortic; paraaortic + ipsi | 4.6 (0.7-12.6) | Grade 1/2:   - Nausea: 34 (67 %) - Diarrhoea: 13 (25%) - Haematologic toxicity: 14 (27%) - Fatigue: 7 (14%)   Grade 3/4:   - Nausea: 1 (2%) - Haematologic toxicity: 4 (8%) | NA | NA | NA |
| Stein | 2014 (1971-2010) | 24 | RT | Paraaortic + iliac | 7 (3.5-23.5) | Mild, dominated by grade 1 nausea and temporary weakness | Peptic ulcer disease: 1 (4%)  Proctitis: 1 (4%)  Chronic abdominal pain: 1 (4%) Neutropenia/thrombocytopenia: 1 (4%) | Pancreatic cancer: 1 (4%) | NA |
| Hallemeier | 2014 (1974-2009) | 251* (52**) | RT | Paraaortic + iliac, M/SC | *15 (0.1-38) | NA | *Peptic ulcer: 13 (5%)  Gastric ulceration: 1 (0.3%)  Small bowel obstruction: 5 (2%) | *Nontesticular GI tract 9 (4%):   - Oesophageal adenocarcinoma: 2 (0.8%) - Gastric adenocarcinoma: 1 (0.3%) - Periampullary adenocarcinoma: 1 (0.3%) - Pancreatic head adenocarcinoma: 1 (0.3%) - Extrahepatic cholangiocarcinoma: 1 (0.3%) - Small bowel adenocarcinoma: 1 (0.3%) - Colonic adenocarcinoma: 2 (0.8%) | 10/20/30y risk of GI SM: 0.5%, 3%, 16% |
| Hu | 2015 (2010-2014) | 4* (3) | RPLND | Modified template, NS | *2.1 (NA) | *Ileus: Clavien Dindo 1: 1 (25%) | none | NA | NA |
| Daneshmand^b^ | 2021 | 55 | RPLND | Open, modified template, NS | NA | 7 (13%):  Clavien Dindo 1-2: 5 (9%):   - Incision ulceration - Bleeding - Ileus requiring nasogastric tube - Emesis/Pain - Low volume ejaculate   Clavien Dindo 3: 2 (4%):   - Chylous ascites - Pulmonary embolism | none | NA | NA |

**Suppl. 4: Toxicities in CSIIA/B seminoma patients according to treatment of studies not included in the systematic review.**

AUC= area under the curve; BCCA= basal cell carcinoma of the skin; BEP= bleomycin/etoposid/cisplatin; Carbo= Carboplatin; CLL= chronic lymphatic leukaemia; CS= clinical stage; CT= chemotherapy; CUP= carcinoma of unknown primary; EP= etoposid/cisplatin; E400P= cisplatin 20mg/m², etoposid 100mg/m²; FU= follow-up; GCNIS= germ cell neoplasia in situ; GI= gastrointestinal; HNSCC= head and neck squamous cell carcinoma; HOP= ifosfamide, vincristine, cisplatin; ipsi= ipsilateral; IQR= interquartile range; M= mediastinal; n= number of patients; NA= not announced; NS= nerve sparing; PVB= cisplatin, vinblastin, bleomycin; RPLND= retroperitoneal lymph node dissection; RT= radiotherapy; SC= supraclavicular; SM= secondary malignancy; VAB= vinblastine, cyclophosphamide, dactinomycin, bleomycin; y= year; *other stages as IIA/B are included; **CS IIC included; ^A^information of 48 patients available; ^B^information of 41 patients available; ^C^25 patients received prophylactic M/SC RT; ^a^results of a very similar patient collective at different follow-up endpoints; ^b^only abstract available
